# Supplementary figures and images for: Effects of an Extract of the Brown Seaweed Ascophylum nodosum on Postprandial Glycaemic Control in Healthy Subjects: A Randomized Controlled Study
Source: Mar Drugs. 2023 May 31;21(6):337. doi: 10.3390/md21060337 (PMC10303464; doi:10.3390/md21060337)

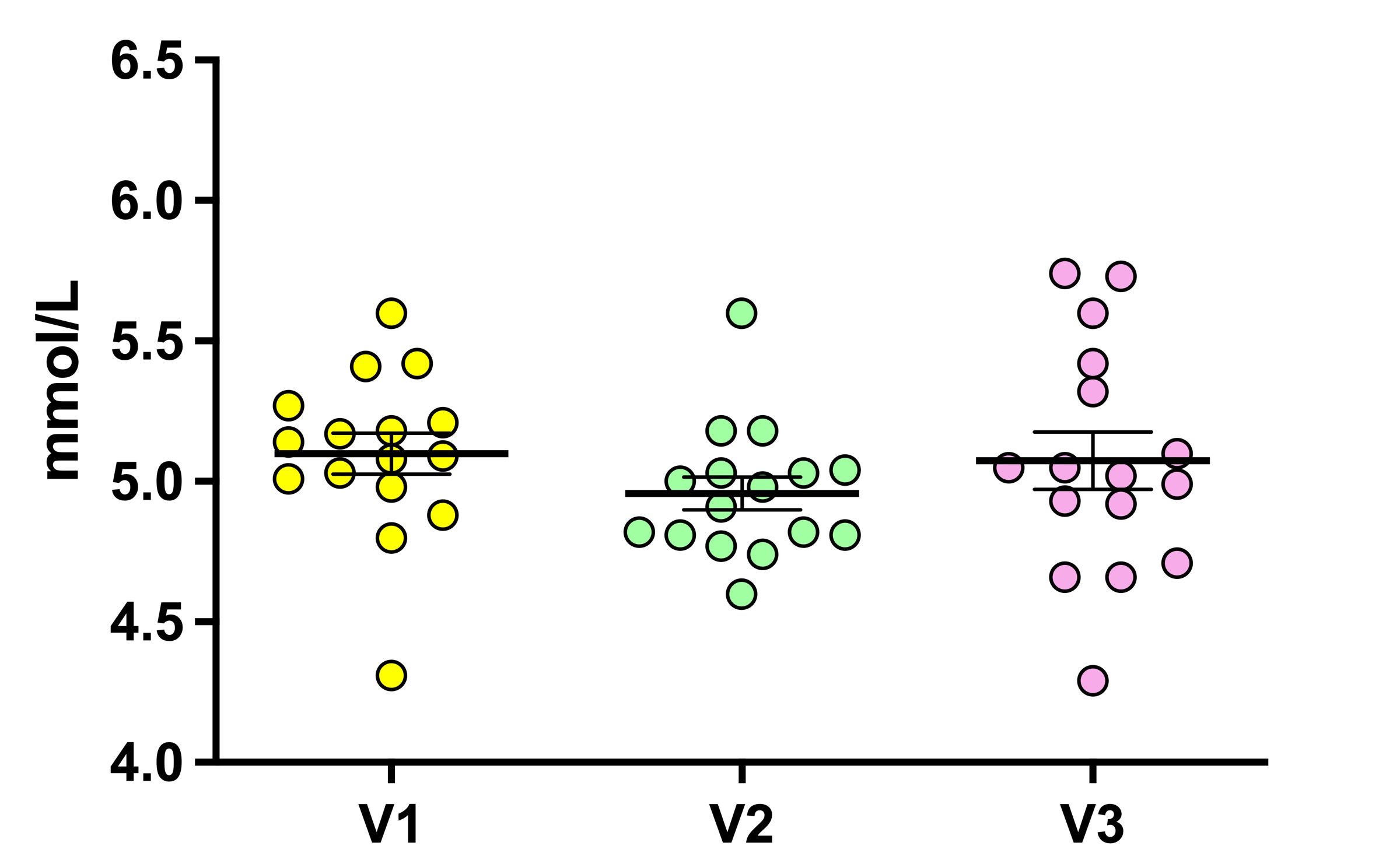

Supplement: Supplementary file 1 [file marinedrugs-21-00337-s001.zip › Figure S1.jpg]

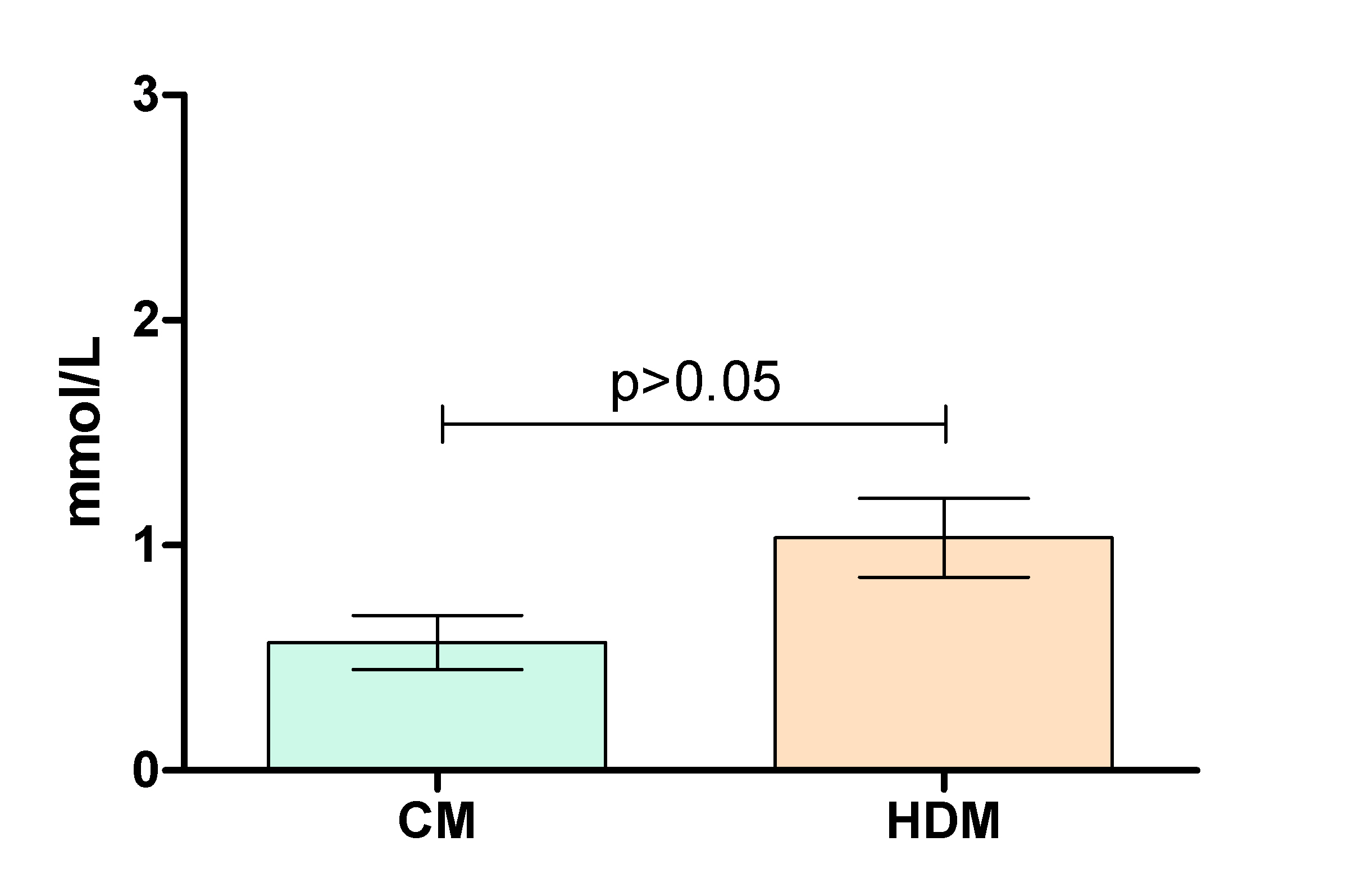

Supplement: Supplementary file 1 [file marinedrugs-21-00337-s001.zip › Figure S2.jpg]

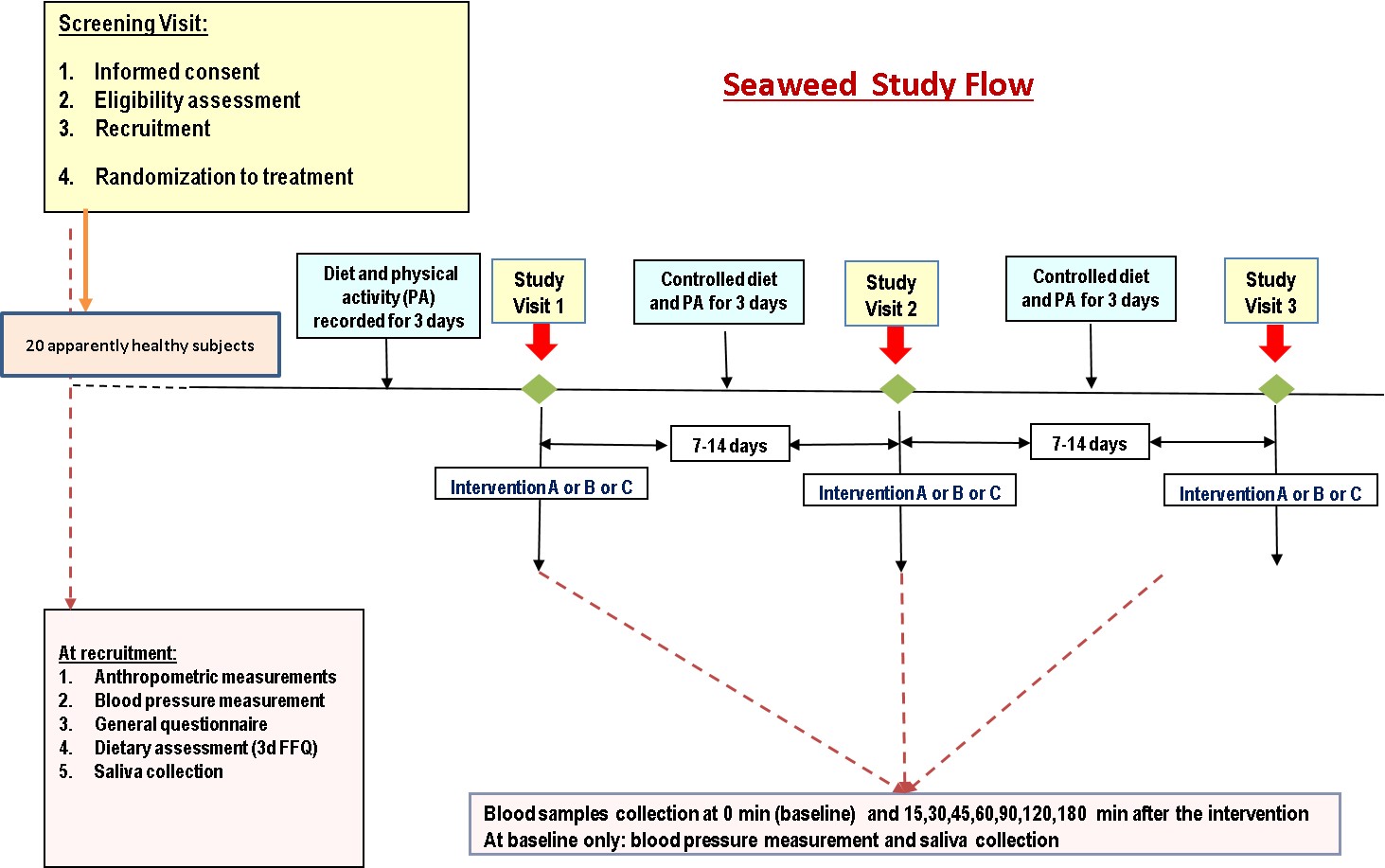

Supplement: Supplementary file 1 [file marinedrugs-21-00337-s001.zip › Figure S3.jpg]

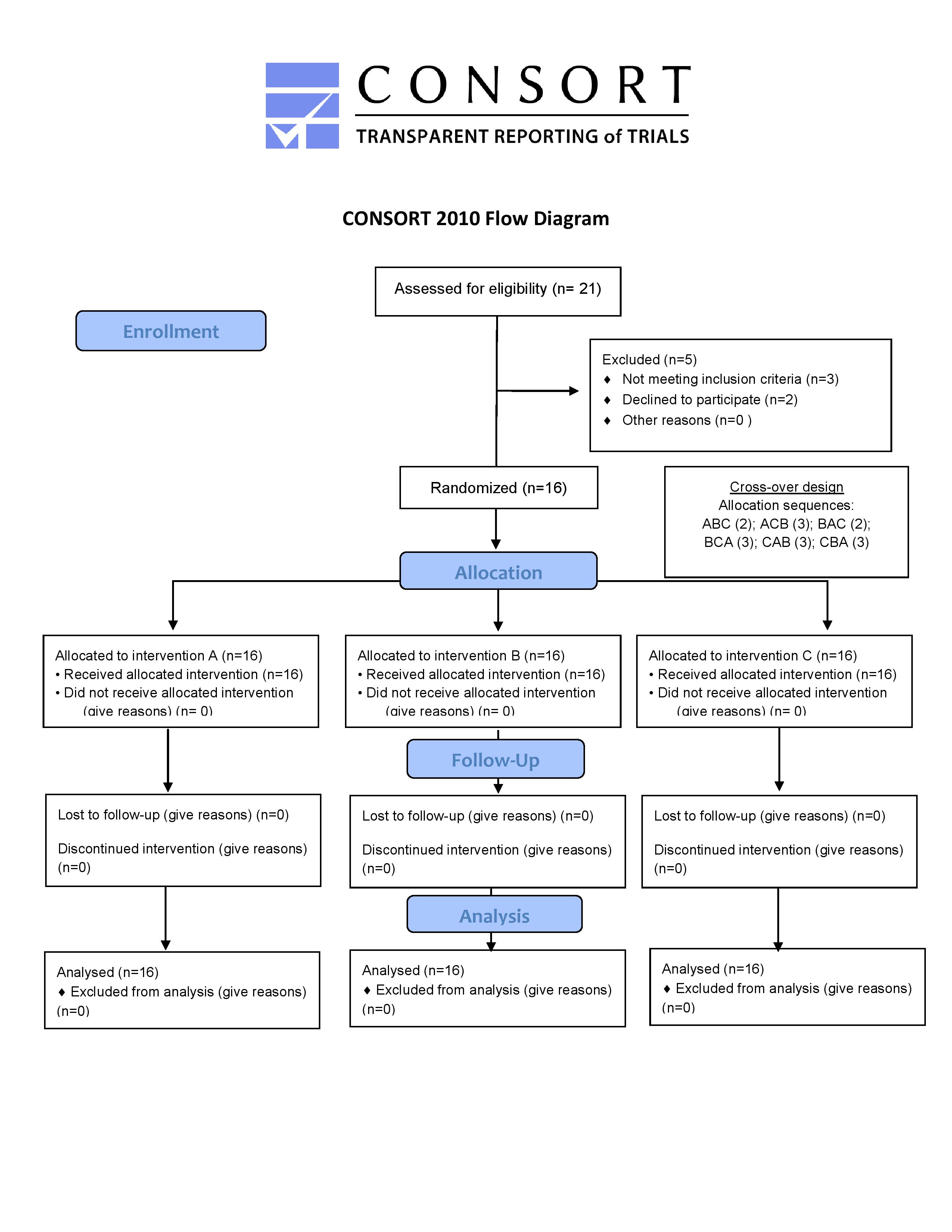

Supplement: Supplementary file 1 [file marinedrugs-21-00337-s001.zip › Figure S4.jpg]
